# Is Follow-Up Endoscopy Necessary in Upper Gastrointestinal Cytomegalovirus Disease?: Erratum CT Findings in Viral Lower Respiratory Tract Infections Caused by Parainfluenza Virus, Influenza Virus and Respiratory Syncytial Virus: Erratum

**DOI:** 10.1097/MD.0000000000006277

**Published:** 2017-02-24

In the articles “Is Follow-Up Endoscopy Necessary in Upper Gastrointestinal Cytomegalovirus Disease?”,^[1]^ which appeared in Volume 95, Issue 19 of *Medicine*, and “CT Findings in Viral Lower Respiratory Tract Infections Caused by Parainfluenza Virus, Influenza Virus and Respiratory Syncytial Virus”,^[2]^ which appeared in Volume 95, Issue 26 of *Medicine*, the Asan Institute of Life Sciences grant number was originally reported incorrectly. The correct grant number in both articles is 2016-462.
